# TTG2-regulated development is related to expression of putative AUXIN RESPONSE FACTOR genes in tobacco

**DOI:** 10.1186/1471-2164-14-806

**Published:** 2013-11-20

**Authors:** Qian Zhu, Baoyan Li, Shuyuan Mu, Bing Han, Runzhi Cui, Manyu Xu, Zhenzhen You, Hansong Dong

**Affiliations:** Plant Growth and Defense Signaling Laboratory, State Ministry of Education Key Laboratory of Integrated Management of Crop Pathogens and Insect Pests, Nanjing Agricultural University, Nanjing, 210095 China; Yantai Academy of Agricultural Sciences, Yantai, 265500 China

## Abstract

**Background:**

The phytohormone auxin mediates a stunning array of plant development through the functions of AUXIN RESPONSE FACTORs (ARFs), which belong to transcription factors and are present as a protein family comprising 10–43 members so far identified in different plant species. Plant development is also subject to regulation by TRANSPARENT TESTA GLABRA (TTG) proteins, such as NtTTG2 that we recently characterized in tobacco *Nicotiana tabacum*. To find the functional linkage between TTG and auxin in the regulation of plant development, we performed *de novo* assembly of the tobacco transcriptome to identify candidates of NtTTG2-regulated *ARF* genes.

**Results:**

The role of NtTTG2 in tobacco growth and development was studied by analyzing the biological effects of gene silencing and overexpression. The *NtTTG2* gene silencing causes repressive effects on vegetative growth, floral anthocyanin synthesis, flower colorization, and seed production. By contrast, the plant growth and development processes are promoted by *NtTTG2* overexpression. The growth/developmental function of NtTTG2 associates with differential expression of putative *ARF* genes identified by *de novo* assembly of the tobacco transcriptome. The transcriptome contains a total of 54,906 unigenes, including 30,124 unigenes (54.86%) with annotated functions and at least 8,024 unigenes (14.61%) assigned to plant growth and development. The transcriptome also contains 455 unigenes (0.83%) related to auxin responses, including 40 putative *ARF* genes. Based on quantitative analyses, the expression of the putative genes is either promoted or inhibited by NtTTG2.

**Conclusions:**

The biological effects of the *NtTTG2* gene silencing and overexpression suggest that NtTTG2 is an essential regulator of growth and development in tobacco. The effects of the altered *NtTTG2* expression on expression levels of putative *ARF* genes identified in the transcriptome suggest that NtTTG2 functions in relation to ARF transcription factors.

**Electronic supplementary material:**

The online version of this article (doi:10.1186/1471-2164-14-806) contains supplementary material, which is available to authorized users.

## Background

TRANSPARENT TESTA GLABRA (TTG) proteins were identified as regulators of trichome and seed development in plants [1–4]. TTGs are characterized by the presence of the WD40 motif [5–7], which refers to the conserved tryptophan (W) and aspartate (D) dipeptide and the length of approximately 40 amino acid residues in a protein sequence [8]. This motif is present as a series of repeats to form protein interaction domain in a variety of proteins [9–11], including all TTGs so far identified except for *Arabidopsis thaliana* AtTTG2, which is a WRKY transcription factor [12]. As the WD40 domain has multiple modes for recruiting different substrates, WD40-containing proteins can serve as interchangeable substrate receptors and interact with diverse types of proteins [11, 13]. Due to this characteristic, TTGs have the potential of regulating both development and defenses in plants [5, 7, 14, 15].

Depending on natures of interacted proteins, TTGs differentiate their functions between development and defense regulations [6, 16]. In *Arabidopsis*, AtTTG1 interacts with the bHLH transcription factor GL3 while recruiting MYB GL1, forming the WD40-bHLH-MYB complex that acts to regulate trichome development [13, 17]. Previously we demonstrated that WD40 enabled tobacco (*Nicotiana tabacum*) TTG1 (NtTTG1) to interact with ParA1 [7], an elicitin protein produced by an oomycete pathogen and inducing hypersensitive cell death in the plant [18]. The NtTTG1-ParA1 interaction was essential for the induction of cell death first in leaf trichomes and then in mesophyll cells [7]. NtTTG2 is an analog of NtTTG1 and both proteins share four WD40 repeats [5, 7, 19]. Recently we showed that NtTTG2 suppressed tobacco resistance to pathogens by indirectly modulating subcellular localization of the NPR1 protein [5], which is a transcriptional regulator of defense responses to pathogens [20–22]. NtTTG2 does not interact with NPR1 but is able to sequester NPR1 from the nucleus, thus preventing NPR1 from transcriptional regulation of defense responses [5]. These studies suggest that TTGs regulate specific processes of development or defenses by either directly or indirectly interacting with other proteins involved in the processes. However, whether NtTTG2 plays a role in development regulation was unclear.

Plant development is subject to a complex regulation of phytohormones with auxin playing a significant role. Auxin mediates a stunning array of the development [23, 24] through the functions of AUXIN RESPONSE FACTOR (ARF) proteins in transcriptional regulation of auxin responses [25–31]. ARF transcription factors constitute a phylogenic protein family [25, 29–32] made of 10–43 members as identified so far in different plant species [33–36]. It is accepted that different members of the ARF protein family target distinct types of auxin-responsive genes, such as those classified in the *Small Auxin Up RNA* (*SAUR*), *Auxin/Indole-Acetic Acid inducible* (*Aux/IAA*), and *Gretchen Hagen 3* (*GH3*) gene families [26, 28, 31, 32, 37–40]. However, only a few of auxin-responsive genes have been shown definitely as ARF targets and to be regulated directly by specific ARFs [28, 31]. In particular, little was known about the regulation of auxin responses in tobacco. In the plant, only four auxin-responsive genes were identified, whereas, at least 29, 134, and 15 members of *Aux/IAA*[23], *SAUR*[41], and *GH3*[42] gene families were characterized in other plants. In tobacco, moreover, only *ARF1*[43] and *ARF8* (JN835423.1) were cloned with full length cDNA sequences while additional eleven *ARF* homologs were identified as expressed sequence tags (ESTs) [44] in contrast to the 10–43 members of the ARF protein family already identified in other plants [33–36]. Therefore, it is rational to conjecture that tobacco may possess more *ARF*s and auxin-responsive genes than the currently known numbers. In addition, there is as yet no study to characterize functions of ARFs in tobacco.

This study was attempted to elucidate the role of NtTTG2 in the growth and development of tobacco and to identify *ARF* gene candidates associated with NtTTG2-regulated growth and development. We show that NtTTG2 is required for the vegetative growth and the development of flowers and seeds. We performed *de novo* assembly of the tobacco *N. tabacum* transcriptome and analyzed functional components in the transcriptome. By using transcriptome information, we identified the unigenes assigned to auxin responses and representing putative *ARF* genes in particular. We present evidence that NtTTG2 affects the expression of putative *ARF* genes in the plant.

## Results

### The repressive effect of NtTTG2 silencing on the vegetative growth and the development of flowers and seeds in tobacco

To elucidate the roles of *NtTTG2* in the growth and development of tobacco *N. tabacum*, we generated the *NtTTG2*-silencing line *TTG2RNAi4* under background of the *N. tabacum* variety NC89 and observed its growth and development in contrast to the *wild-type* (*WT*) phenotype. The transgenic NC89 line *WT RFP* was used as a control since it highly resembled *WT* in the development process (Additional file 1: Figure S1a) and biological productivity (Additional file 1: Figure S1b,c). *RFP* is a red-fluorescent protein-encoding gene used in detecting subcellular localization of the protein [5] but is not related to this study. Molecular and genetic characterizations of transgenic plants have been documented in a recently published article [5].

**Figure 1:**
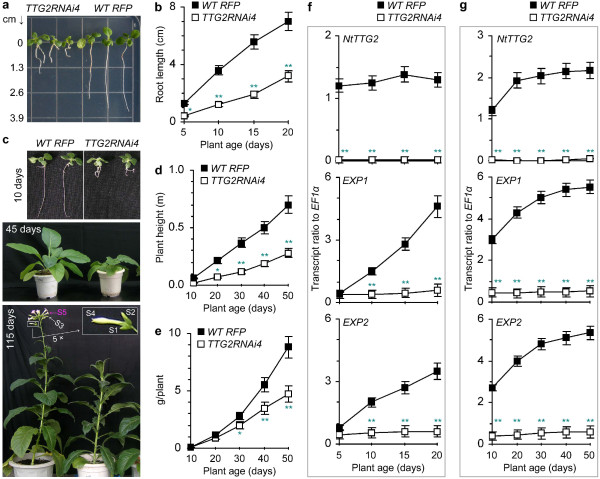
**The effect of** ***NtTTG2*** **silencing on tobacco growth.** The *NtTTG2*-silenced tobacco line *TTG2RNAi4* was compared to the control *WT RFP* plant in terms of the vegetative growth and the expression of *NtTTG2* and *EXP* genes. **(a, b)** Root monitoring after 10-day growth on the medium. **(c)** Plants grown in pots. Floral development stages S1–S5 are indicated. **(d, e)** The biomass of plants from **(c)**. **(f)** Real-time RT-PCR analyses of the indicated genes expressed in roots of plants from **(b)**. **(g)** Real-time RT-PCR analyses of gene expression in leaves of 45-day-old plants grown in pots and equivalent to those in **(c)**. In **(b, d-g)**, data shown are mean values ± standard deviation (SD) bas of results from five experimental repeats (50 plants/repeat). Data were analyzed by the ANOVA method along with one-way Fisher’s Least Significant Difference (LSD) test. Significant differences between data pairs at the corresponding time points are indicated by single asterisks (*P* < 0.05) or double asterisks (*P* < 0.01) on the graphs.

In this study, we investigated the vegetative growth of *WT RFP* and *TTG2RNAi4* plants on Murashige and Skoog (MS) agar medium and in pots. We also analyzed the expression of the *NtTTG2* gene along with the expansin (EXP) protein-encoding genes *EXP1* and *EXP2*, which are responsive to auxin and contribute to plant growth [45, 46]. When plants were grown on the medium, the root length of *TTG2RNAi4* was much shorter than that of *WT RFP* (Figure 1a), and this difference was statistically significant (*P* < 0.05 or 0.01) as scored in 5–20 days (Figure 1b). Similar results were observed on plants grown in pots (Figure 1c), and compared to *WT RFP*, *TTG2RNAi4* was significantly (*P* < 0.05 or 0.01) impaired in the vegetative growth based on the biomass scored as height (Figure 1d) and weight (Figure 1e) of fresh plants. Consistently, the expression of *NtTTG2*, *EXP1*, and *EXP2* genes was detected at substantial levels in both roots (Figure 1f) and leaves (Figure 1g) of *WT RFP*, but little in the *TTG2RNAi4* plant. These analyses indicate that *NtTTG2* is required for the vegetative growth of tobacco in correlation with the expression of *EXP1* and *EXP2* in the plant.

**Figure 2:**
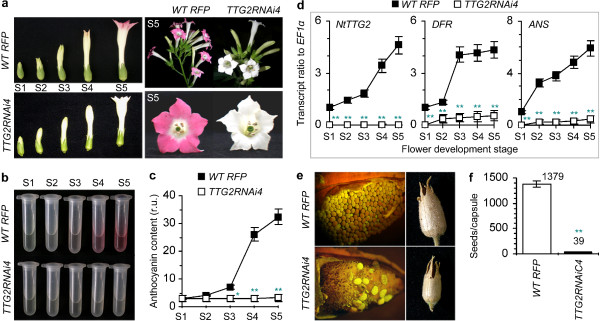
**The effects of** ***NtTTG2*** **silencing on flower and seed development. (a)** Flowers at five development stages (S1-S5) from 120-d-old *WT/RFP* and 160-d-old *TTG2RNAi4* plants. **(b, c)** Flower anthocyanin extracts and spectrophotometric analyses. The floral anthocyanin content was quantified as relative unit (r.u.). **(d)** Real-time RT-PCR analyses of floral transcripts of the *NtTTG2* gene as well as the *DFR* and *ANS* genes, which are involved in the late stage of the anthocyanin biosynthesis pathway. **(e)** Growing fruits (left) from 140-d-old *WT/RFP* and 170-d-old *TTG2RNAi4*, and mature fruits (right) from 170-d-old *WT/RFP* and 210-d-old *TTG2RNAi4* plants. **(f)** The number of seeds per mature fruit. Data were given as mean values ± SD bars of results from three **(c, d)** and five **(f)** experimental repeats (30 fruits/repeat). Significant levels (*P < 0.05; **P < 0.01) in differences between corresponding data pairs were analyzed by one-way ANOVA and LSD test.

Then, we observed the flower and seed development, which has been shown to be regulated by TTG proteins in *Arabidopsis*[16] and petunia [2, 4]. We analyzed the effects of *NtTTG2* silencing on the floral development throughout stages S1-S5 (Figures 1c and 2a). Average time to S1 was 105 and 143, and average time from S1 to S5 was 7 and 9 days in *WT RFP* and *TTG2RNAi4* plants, respectively. The effect of *NtTTG2* silencing on flower color was marked. Plants produce anthocyanin, a water-soluble pigment that may make flowers differently colored [47]. *WT RFP* flowers had visible anthocyanin production since S4 and petals appeared red at S5 (Figure 2a). Floral anthocyanin extracts (Figure 2b) and spectrophotometric analyses (Figure 2c) indicated that *WT RFP* flowers started to produce anthocyanin at S3 and that abundant amounts of the pigment were detected at S4 and S5. By contrast, little anthocyanin was found in *TTG2RNAi4* flowers (Figure 2a-c).

Next, we correlated the variation of anthocyanin production with the expression of the *DFR* and *ANS* genes involved in late stage of the anthocyanin biosynthesis pathway that mainly comprises nine genes [48–50]. The floral expression of *DFR* and *ANS* was *NtTTG2*-dependent (Figure 2d) but that of the other genes was not (Additional file 2: Figure S2). In *WT RFP*, the expression of *NtTTG2*, *DFR*, and *ANS* was increased with the floral development, but the three genes were little expressed in *TTG2RNiAi4* (Figure 2d). Therefore, *NtTTG2* was required for *DFR* and *ANS* expression with consequent effects on anthocyanin production and flower colorization (Figure 2a-c). In addition, *NtTTG2* was also required for seed production as *NtTTG2* silencing caused acute seed abortion (Figure 2e), and more than 97% seeds were aborted in *TTG2RNAi4* compared to *WT RFP* (Figure 2f). These analyses indicate that *NtTTG2* functions in flower and seed development of tobacco.

### The promoting effect of NtTTG2 overexpression on tobacco growth and development

**Figure 3:**
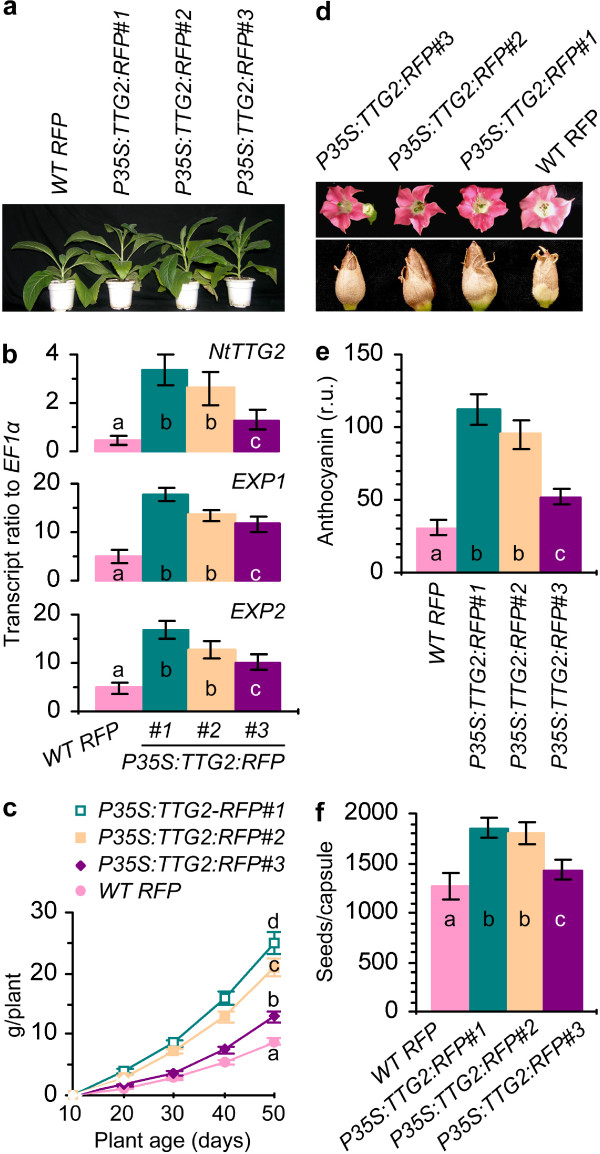
**The effects of** ***NtTTG2*** **overexpression on main characters of tobacco development. (a)** Forty-day-old plants. **(b)** Real-time RT-PCR analyses of *EXP1* and *EXP2* expression in leaves of plants from **(a)**. **(c)** The biomass of plants from **(a)**. **(d)** S5 flowers and mature seeds. **(e)** S5 flower anthocyanin content given as relative unit (r.u.). **(f)** The number of seeds per mature fruit. Quantitative data are presented as mean values ± SD bars of results from five experimental repeats each involving 15 plants **(b, c)**, 30 flowers **(e)**, or 30 fruits **(f)**. Different letters on curve and bar graphs indicate significant (*P* < 0.01) differences by one-way ANOVA and LSD test.

To further demonstrate the role of *NtTTG2* in tobacco growth and development, we generated the *NtTTG2*-overexpressing (*P35S:TTG2:RFP*) NC89 lines [5] and observed their growth and development characters in comparison with the *WT RFP* plant. Here, *P35S* refers to the cauliflower mosaic virus 35S promoter. In all tested characters, *P35S:TTG2:RFP* lines were opposite to *TTG2RNiAi4*. Compared to *WT RFP*, *P35S:TTG2:RFP* plants grew better (Figure 3a) and had higher levels of *EXP* expression (Figure 3b) and biomass (Figure 3c). *P35S:TTG2:RFP* plants also had better flower colorization (Figure 3d), higher levels of *NtTTG2*, *DFR*, or *ANS* expression from S1 through S5 (Additional file 2: Figure S2), and more anthocyanin production in S4 flowers (Figure 3e). Moreover, *P35S:TTG2:RFP* capsules were well developed (Figure 3d) with more seeds per fruit (Figure 3f). Significant (*P* < 0.01) differences were found between *WT RFP* and each line of *P35S:TTG2:RFP* while *P35S:TTG2:RFP#1* was the best in these developmental characters. Clearly, *NtTTG2* overexpression causes a promoting effect on the vegetative growth and the development of flowers and seeds in tobacco.

### Transcriptome sequencing and de novo assembly

We sought to identify *ARF* gene candidates that might associate with NtTTG2-regulated growth and development in the tobacco *N. tabacum* variety NC89. We performed *de novo* assembly of the transcriptome by using the RNA-Seq technique and a mixture of equal quantities of total RNA isolated from leaves of *WT RFP*, *TTG2RNAi4*, and *P35S:TTG2:RFP#1*. The mixed RNA sample from three biological repeats was used in the library construction and the transcriptome sequencing with the Illumina HiSeq 2000 sequencer, followed by *de novo* assembly with the Trinity assembler.

**Figure 4:**
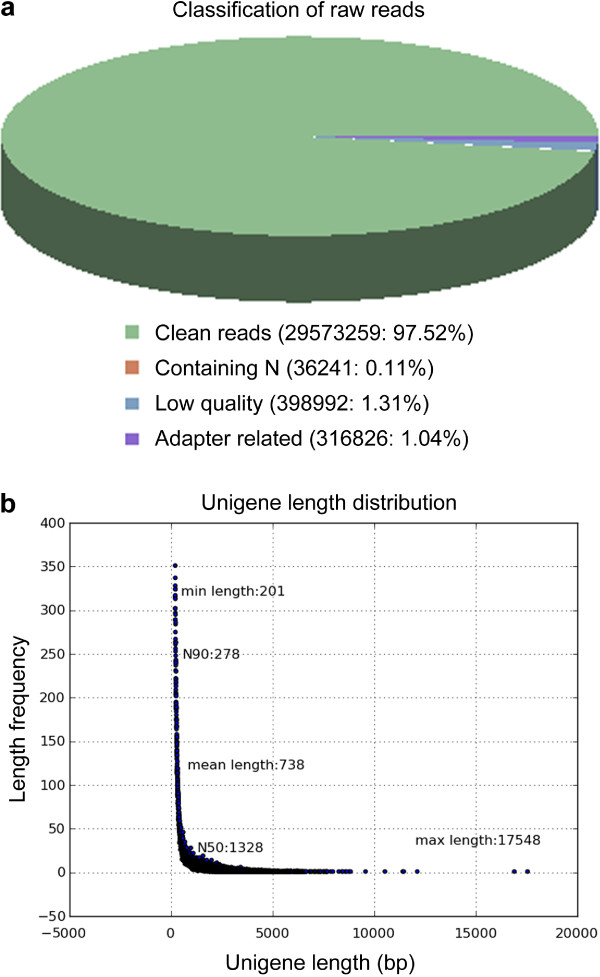
**Assessment of the transcriptome assembly quality. (a)** Components of raw reads. The character N in the “Containing N” type indicate that nucleotides were not recognized. Different colors represent components. **(b)** Unigene length distribution.

In all, 30,325,328 raw reads were generated, and a total of 29,573,259 clean reads, occupying 97.52% of total, were obtained after three types of reads (containing adaptor sequences, more than 10% unknown bases, and more than half of the quality values of the bases were lower than 5) were removed by Illumina RNA-Seq deep-sequencing (Figure 4a). Using the high quality reads, 111,207 transcripts were assembled with an average length of 1,031 bp, a length variation in 201–17,548 bp, and 15,824 (14.23%) transcripts longer than 2,000 bp. Redundancy rate in 111,207 transcripts was 50.63%, and the longest copy of redundant transcripts was regarded as a unigene [51, 52]. In total, 54,906 unigenes were identified in the transcriptome and their length was found to vary from 201 bp to 17,548 bp (Figure 4b). The length of 33,116 (60.31%), 9,727 (17.72%), and 7,617 (13.87%) unigenes ranges accordingly in 201–500, 501–1,000, 1,001-2,000 bp while 4,446 (8.10%) unigenes are longer than 2,000 bp.

### Functional annotation of unigenes

**Figure 5:**
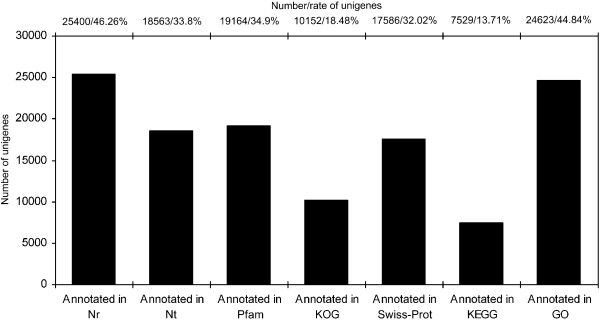
**Distributions of unigenes in seven public databases.**

**Figure 6:**
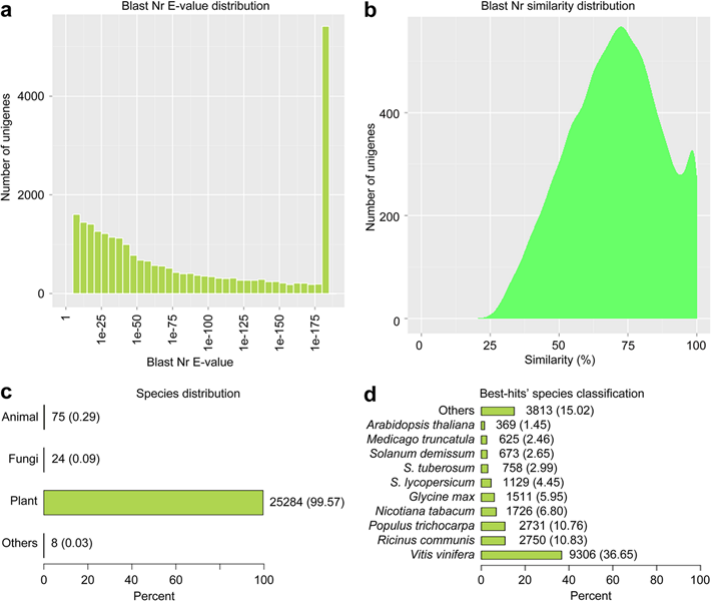
**Characteristics of similarity search against Nr databases. (a)** E-value distribution of BLAST hits for unigenes with a cutoff E-value of 1.0E-5. **(b)** Similarity distribution of the top BLAST hits for unigenes. **(c, d)** Organism species distribution of the top BLAST hits for unigenes in Nr database. The numbers of unigenes are indicated together with percentages placed in parentheses.

Sequences of assembled unigenes were searched against seven different databases (Figure 5) with an E-value threshold of 10^-5^ (1.0E-5). As shown in Figure 5, various numbers of unigenes showed significant similarity to the lodged in those databases; similar sequences of 30,124 (54.86%) unigenes were found in at least one database while 4,009 (7.3%) unigenes shared similarity to sequences deposited in all databases. However, sequence orientation of 24,782 (45.14%) unigenes is still unknown. Based on the E-value distribution of the top hits, 57.45% of the mapped unigene sequences showed significant homology (less than 1.0E-50) with genes deposited in the Nr (NCBI non-redundant protein sequences) database (Figure 6a). By searching against the same database, 86.69% and 40.82% of the unigenes were found to possess sequence similarities greater than 50% and 75%, respectively (Figure 6b). In regard to organism sources of homologs, approximately 99.57% of unigenes with Nr annotations were matched to plant genes (Figure 6c). While 10.83%, 10.76%, 6.80%, 5.95%, 36.65% of the mapped unigene sequences possess significant similarity, respectively with orthologs of *Ricinus communis*, *Populus trichocarpa*, *Nicotiana tabacum*, *Glycine max*, and *Vitis vinifera* (Figure 6d).

### Functional classification of unigenes

**Figure 7:**
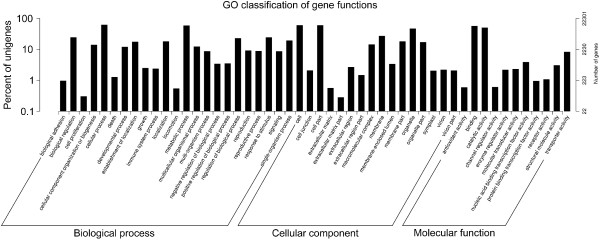
**GO classifications of assembled unigenes.**

Functions of the unigenes were classified with GO (Gene Ontology), an international standardized gene functional classification system that defines genes in three ontologies: “molecular function”, “cellular component”, and “biological process”. We used the Blast2GO program [53] to obtain GO annotation of the unigenes in the Nr database. Then, we used the WEGO software [54] to classify the unigenes to GO function terms. In total, 24,623 unigenes (44.85%) have GO annotation, and these unigenes are assigned to 58 functional terms (Figure 7). Of these 24,623 unigenes, the “biological process” occupied the majority (89,767, 46.14%), followed by “cellular component” (71,929, 36.97%) and “molecular function” (32,846, 16.88%). Around the comprehensive range of GO categories, “cellular process” (16,726, 8.60%), “cell” (15,739, 8.10%), “cell part” (15,680, 8.06%), “metabolic process” (15,460, 7.95%), and “binding” (14,641, 7.53%) proteins are in the top five rank and make up the majority of the unigenes (Figure 7). “Cell proliferation”, “extracellular matrix part”, and “nucleotide proteins” (not shown in Figure 7 due to small values) together occupy only 0.92% of unigenes that have GO annotation. In particular, numbers of unigenes are 614 (2.49% of total number of GO-annotated genes), 2,943 (11.95%), 2,277 (9.25%), and 2,190 (8.89%) assigned to “growth”, “development process”, “reproduction”, “reproductive process” in the biological process category (Figure 7). Moreover, 3,248 (13.19%) unigenes fall into the functional term of transcription factor activities in the “molecular function category” (Figure 7).

**Figure 8:**
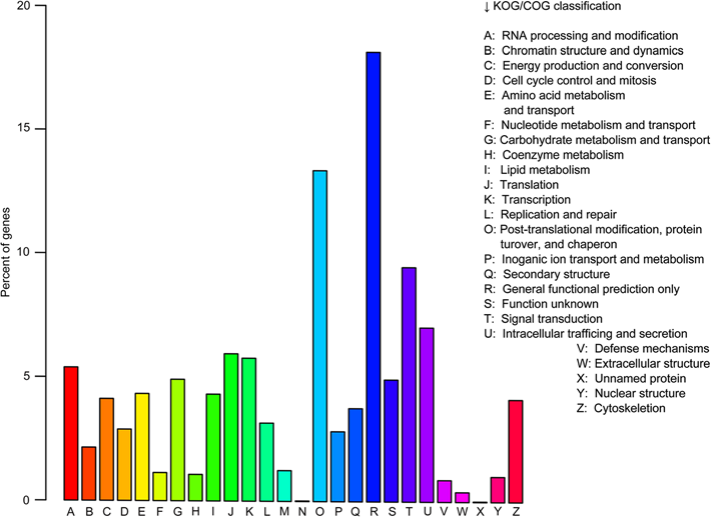
**KOG/COG classification of unigenes.**

Functions of the unigenes were also classified with the KOG/COG (euKaryotic Ortholog Groups/Clusters of Orthologous Groups of Proteins) databases that classify predicted unigene products into distinct functional groups [55]. By the KOG/COG analysis, 10,152 of the unigenes were assigned to the 26 COG classifications (Figure 8). Some single unigenes were assigned to several COG categories, and this resulted in a record of 11,439 unigenes in all COG classifications. In comparison, the category of “General function prediction” contains the greatest number of unigenes (1,874, 16.38%), followed by “Post-translational modification, protein turnover, and chaperon” (1,346, 11.77%), “Signal Transduction” (970, 8.48%), “Intracellular trafficking and secretion” (692, 6.05%), “Transcription” (627, 4.48%), and “RNA processing and modification” (539, 4.72%).

### Metabolic pathway analysis of unigenes

**Figure 9:**
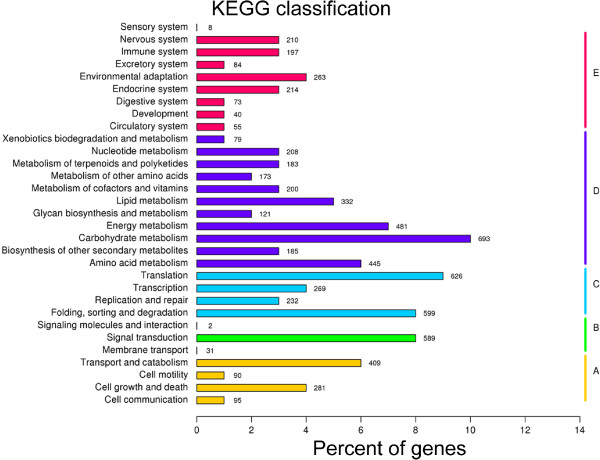
**KEGG classification of unigenes.** Capital letters back to the colored bars indicate five main categories: **A**, Cellular Processes; **B**, Environmental Information Processing; **C**, Genetic Information Processing; **D**, Metabolism; and **E**, Organism Systems.

Orientation of unigenes in metabolic pathways was analyzed with the KEGG (Kyoto Encyclopedia of Genes and Genomes) database, which integrates genomic, biochemical, and functional information in various cellular processes [56]. Based on BLASTx searches with an E-value threshold of 10^-5^, 7,529 of the 54,906 unigenes were assigned to 31 KEGG pathways in the KEGG database (Figure 9). Those pathways were divided into five branches with numbers/percents of unigenes shown in parentheses: “Cellular Processes” (902/1.64%), “Environmental Information Processing” (658/1.20%), “Genetic Information Processing” (1,830/3.33%), “Metabolism” (3,255/5.93%), and “Organismal Systems” (1,192/2.17%). Of the 31 KEGG pathways, moreover, the pathway of “carbohydrate metabolism” contains homologs of the most unigenes (724/1.32%), followed by “translation” (665/1.21%), “folding, sorting and degradation” (625/1.14%), “signal transduction” (622/1.13%), “energy metabolism” (506/0.92%), and “amino acid metabolism” (469/0.85%), whereas, only no more than 10 unigenes were assigned to “signaling molecules and interaction” and “sensory system”.

### Unigenes assigned to auxin responses and the effects of NtTTG2 on the expression of putative ARF genes

**Figure 10:**
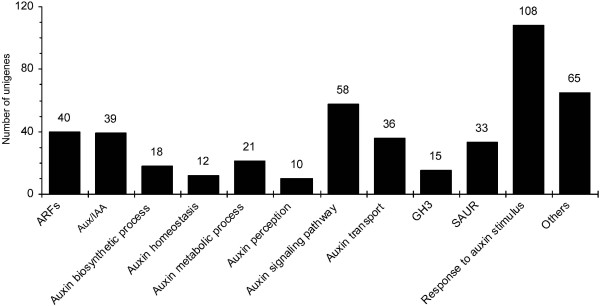
**Phylogenic protein families involved in auxin responses and identified in the transcriptome.** The number of genes in the corresponding protein family group (bottom character panel) is shown on top of the bar graph. The “Others” group mainly contains auxin-repressed and auxin-induced proteins that do not belong to any of the designated groups.

**Figure 11:**
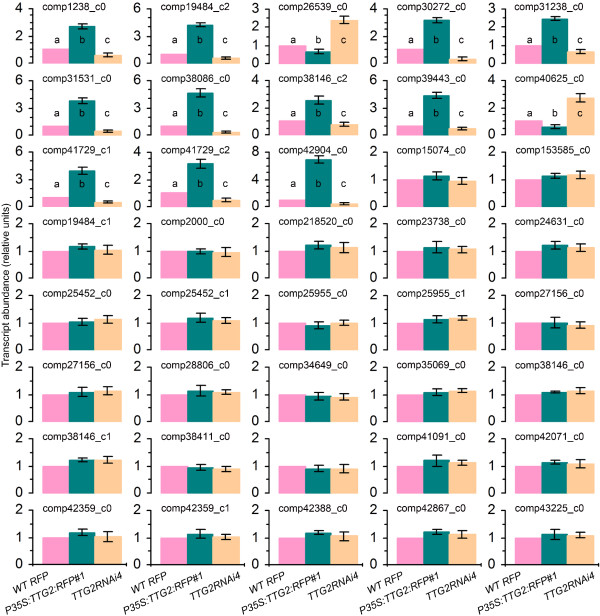
**The effects of NtTTG2 on the expression of putative** ***ARF*** **genes.** The effects were determined by analyzing the expression of putative *ARF* genes in *WT RFP*, *P35S:TTG2:RFP#1*, and *TTG2RNAi4* plants. Putative *ARF* genes are shown as identification code in the transcriptome inventory. Gene expression levels in the control plant were regarded as 1 to qualify relative amounts of the same gene transcripts in the other phenotypes of plant. Data shown are mean values ± SD bars of results from three experimental repeats (15 plants/repeat). Different letters on bar graphs indicate significant (*P* < 0.01) differences by one-way ANOVA and LSD test.

Browsing of the tobacco transcriptome profile identified 455 unigenes assigned to the regulation of auxin responses in plants (Figure 10a). In particular, 40, 39, 15, and 33 unigenes were predicted to be *ARF*, *Aux/IAA*, *GH3*, and *SAUR* gene candidates while additional 10, 58, and 108 unigenes were categorized as auxin perception, auxin signaling pathway, and response to auxin stimulus, respectively. This suggests that the tobacco transcriptome is suited for a potential use in further studies to characterize the regulation of auxin responses in the plant. To examine this notion, we analyzed the effects of NtTTG2 on the expression of the 40 putative *ARF* genes (Additional file 3: Table S1) by testing their expression in *WT RFP*, *TTG2RNAi4*, and *P35S:TTG2:RFP#1* plants. As shown in Figure 11, expression levels of 27 putative *ARF* genes were similar but the other 13 candidates were expressed at different extents in the three genotypes of tobacco. Of the 13 differently expressed putative *ARF* genes, two (comp26539_c0 and comp40625_c0) were downregulated by NtTTG2 as their transcript abundances were decreased in *P35S:TTG2:RFP#1* but increased in *TTG2RNAi4*, compared to *WT RFP* used as a transgenic control plant. Inversely, the other 11 putative *ARF* genes (comp1238_c0, comp19484_c2, comp30272_c0, comp31328_c0, comp31531_c0, comp38086_c0, comp38146_c2, comp39443_c0, comp41729_c1, comp41729_c2, and comp42904_c0) were upregulated by NtTTG2, and the expression of these putative genes was enhanced in *P35S:TTG2:RFP#1* but suppressed in *TTG2RNAi4*, compared to the control plant. Therefore, NtTTG2 indeed affects the transcript abundance of a relatively small number of *ARF* gene candidates and the effect is either positive or negative depending on particular candidates.

## Discussion

The repressive effect of the *NtTTG2* gene silencing and the promoting effect of the gene overexpression on vegetative growth, floral anthocyanin synthesis, flower colorization, and seed production (Figures 1, 2 and 3; Additional file 1: Figure S1, Additional file 2: Figures S2) suggest that NtTTG2 is an essential regulator of the growth and development processes in tobacco *N. tabacum*. The growth/developmental role of *NtTTG2* associates with its effects on the expression of *EXP*, *DFR*, and *ANS* genes, which are essentially involved in the plant growth and development pathways [6, 48, 57, 58].

EXPs have unique “loosening” effects on plant cell walls [58] and promote cell expansion [58–61] and plant growth [60, 62, 63] under regulation by phytohormones including auxin [64, 65]. Of six *EXP* genes identified in tobacco and hundreds of *EXP*s in other plants ([57, 58, 66, 67]; http://www.personal.psu.edu/fsl/ExpCentral]), *EXP1* and *EXP2* are widely characterized to be required for plant growth [57, 58, 62–64]. *EXP1* and *EXP2* expression consistent with the biomass (Figures 1 and 3) suggests that both *EXP*s contribute to *NtTTG2*-mediated vegetative growth of tobacco. Since the expression of *EXP*s is subject to multiple regulations [64, 65], it is not surprising that *NtTTG2* silencing reduces but does not eliminate *EXP* expression.

NtTTG2 differently affects the expression of genes involved in the anthocyanin biosynthesis pathway (Figures 2 and 3; Additional file 2: Figure S2). NtTTG2 promotes the expression of the *DFR* and *ANS* genes, which function at the penultimate and antepenultimate steps, respectively in the anthocyanin biosynthesis pathway that mainly involves nine genes [6, 50]. *DFR* encodes dihydroflavonol 4-reductase that reduces three dihydroflavonols to three leucoanthocyanidins [6, 60, 68], and then two leucoanthocyanidins are converted by *ANS*-encoded anthocyanin synthase to anthocyanidin [69]. The other seven *NtTTG2*-independent genes (Additional file 2: Figure S2) encode enzymes with specific actions on single substrates [6, 50]. Therefore, *NtTTG2* affects anthocyanin production and flower colorization through affecting the expression of genes critical to the final formation of anthocyanin (Figures 2 and 3; Additional file 2: Figure S2). In addition, severe abortions owing to *NtTTG2* silencing and marked promotion of the seed production by the gene overexpression suggest that *NtTTG2* plays a role in seed development (Figures 3 and 5). How NtTTG2 regulates the expression of *DFR* and *ANS*, and *EXP*s as well, remains to be studied.

To reveal the molecular mechanism that underlies the growth/developmental role of *NtTTG2*, we performed *de novo* assembly of the tobacco *N. tabacum* transcriptome to identify *NtTTG2*-affected *ARF* gene candidates in the case that little were known about the regulation of auxin responses in tobacco [40, 41, 43]. The transcriptome was assembled with a high quality based on proportion of clean reads in total raw reads and length sorting of unigenes (Figure 4). High quality of the transcriptome assembling can be also found in significant homology of unigene sequences with those searched from the public databases (Figures 5 and 6). Analyses of the transcriptome have created substantial data on functional components in the plant genome: a total of 54,906 unigenes, 30,124 unigenes (54.86%) with annotated functions, at least 8,024 unigenes (14.61%) assigned to plant growth and development, and 455 unigenes (0.83%) related to the regulation of auxin responses (Figures 5, 7, 8 and 9) including 40 putative *ARF* genes (Figure 10; Additional file 3: Table S1). In addition, 24,782 (45.14%) unigenes do not have annotation in any of the databases.

With such substantial genetic components, the *N. tabacum* transcriptomics inventory should be useful in further studies on a broad perspective of the plant genetics and on physiological or biochemical connections between TTGs and auxin responses in particular. In support of this notion, we show that NtTTG2 significantly affects transcript abundances of 13 of 40 putative *ARF* genes and the effect is either positive or negative depending on particular unigenes (Figure 11). This indicates that NtTTG2 may function in association with ARF transcription factors in regulating the plant growth and development. In a recently published draft genome sequence of tobacco *N. benthamiana* (http://solgenomics.net/organism/Nicotiana_benthamiana/genome), 24 immunity-associated genes were retrieved from approximately 16,000 unigenes and this was regarded as evidence for the usefulness of the genome assembly in the study of plant-pathogen interactions [70]. In comparison, the effects of NtTTG2 on transcript abundances of putative *ARF* genes (Figure 11) suggest that the *N. tabacum* transcriptome is useful to the study of auxin responses in the plant.

However, the current technique used in *de novo* transcriptome assembling is unable to obtain full-length sequences of most unigenes. Instead, most unigenes are identified based on partial sequences present in the transcriptome. In this regard, it is necessary to perform a comprehensive comparison between the 54,906 unigenes identified in the *N. tabacum* transcriptome and informative genomic sequence of the plant. The *N. benthamiana* genome sequence was established by assembling of 461,463 sequences with an average length of 5,336 bp [70]. In the current study, the *N. tabacum* transcriptome was assembled from 111,207 transcripts with an average length of 1,031 bp. Thus, it is rational to speculate that unigenes identified in this study must be much shorter than the actual length of real genes. Due to this reason, further characterizing these unigenes is a great challenge.

It is also a great challenge to characterize the functional relationship between NtTTG2 and ARFs in tobacco. Full-length sequences of the putative *ARF* genes remain unknown except for comp42904_c0, which is 100% identical with the sequence of the *ARF8* gene from the *N. tabacum* variety NC89 (JN835423.1). The predicted ARF8 protein is characteristic of transcription activators based on glutamine abundance in the middle region of the protein sequence, in contrast to serine abundance in the same region of an ARF transcription repressor [25]. Unfortunately, information available from the *N. tabacum* transcriptome is insufficient to show whether the 39 *ARF* candidates encode transcription activators or repressors. Cloning of full-length sequences of the *ARF* candidates, analyses of their roles as transcription activators or repressors, and characterization of their functional relationships with NtTTG2 will be the subject of further studies.

## Conclusions

Molecular and genetic analyses performed in this study demonstrate that *NtTTG2* plays an indispensable role in the regulation of growth and development processes in tobacco *N. tabacum*. The growth/developmental role of *NtTTG2* associates with its function in regulating the expression of growth-promoting *EXP* genes as well as the *DFR* and *ANS* genes essentially involved in the anthocyanin biosynthesis pathway. In the growth/developmental regulation, moreover, *NtTTG2* may have a functional linkage with auxin responses as indicated by the effects of *NtTTG2* silencing and overexpression on the expression of 13 putative *ARF* genes identified in the tobacco transcriptome assembled by the *de novo* assembly method. Substantial data of genetic components derived from analyses of the transcriptome should be useful for studies in the future on the plant genetics in general and on the functional relationship between NtTTG2 and auxin in regulating growth and development of the plant in particular.

## Methods

### Plant material and growth conditions

The WT and transgenic plants of the tobacco (*N. tabacum*) variety NC89 were used in this study. Transgenic plants included the *NtTTG2*-silencing line *TTG2RNAi4*, *NtTTG2*-overexpressing *P35S:TTG2:RFP* lines, and transgenic control line *WT RFP* generated previously [5]. Seedlings used in monitoring of root growth were grown on MS agar medium in 10-cm square plates incubated in an environment-controlled chamber with a 14-hour-light (250 μE/m^2^/s at 26°C) and 10-hour-dark (23°C) cycle. Plants used in developmental observations were grown a mixture of sand and potting soil in 360-mL pots incubated in a greenhouse at 23–26°C.

### Plant growth, development, and anthocyanin analyses

The vegetative growth and development of flowers and seeds were monitored. Total anthocyanin was extracted by homogenizing fresh flowers in liquid nitrogen. Fine flower powders were immediately lyophilized and maintained at −80°C until use. Total anthocyanin in lyophilized flower powders was extracted by incubation in methanol solution containing 1% hydrochloric acid for 18 h at room temperature and under moderate shaking. The extract suspension was centrifuged (12,000 g, 4°C, 1 min) to precipitate cellular debris and collect supernatant. Anthocyanin concentration in the supernatant was quantified by spectrophotometry and the endogenous content was scored in contrast to fresh weight of flowers used in the extract preparation.

### RNA isolation and quality verification

Total RNA used in gene expression analysis was isolated from the fourth youngest leaves of 40-day–old plants grown in ports and S1-S5 flowers or 15-day-old immature fruits (capsules) of proper plants grown in pots. Total RNA used in the transcriptome assembly was isolated from the fourth youngest leaves of 40-day-old plants grown in pots. In order to eliminate the variation between individual plants, three biological repetitions were performed and in each repetition, RNA was isolated from a mixture of five leaves each from an individual plant. The collected leaves were homogenized in liquid nitrogen. RNA was isolated from frozen leaf powders were performed by using TRIzolH Reagent (Invitrogen, San Diego, USA) and then treated with DNase I (Invitrogen). The quality of RNA samples was verified using a 2100 Bioana-lyzer RNA Nanochip (Agilent, Santa Clara, CA) with the criteria of RIN (RNA Integrity Number) greater than 8.5 and 28S:18S greater than 1.5. Concentrations of aqueous RNA preparations were determined by spectrophotometry at the standard of OD_260/280_ between 1.8 and 2.2 along with OD_260/230_ greater than 1.8.

### Gene expression analysis

Gene expression was determined by real-time reverse transcriptase-polymerase chain reaction (RT-PCR) using specific primers (Additional file 4: Tables S2 and Additional file 5: Table S3) and previously described protocols [71, 72]. The constitutively expressed *EF1α* gene was used as a reference [62, 63, 71–73]. The expression level of a tested gene was quantified as the transcript ratio to *EF1α*.

### cDNA library construction and sequencing

Equal quantities of total RNA from three samples were mixed to prepare the pooled RNA sample for RNA-Seq. A total amount of 30 μg mixed RNA sample confirmed for RIN values above 8.0 was used as input material in constructing the sequencing library. The library was generated using Illumina TruSeq™ RNA Sample Preparation Kit (Illumia, San Diego, USA) as per the manufacturer’s recommendations and a tetrad index code was added to the sample for subsequent documentation. High quality mRNA was obtained by purification of the total RNA sample using poly-T oligo-attached magnetic beads. Fragmentation was carried out using divalent cations under elevated temperature in Illumina proprietary fragmentation buffer. First strand cDNA was synthesized using random oligonucleotides and SuperScript II. Second strand cDNA synthesis was subsequently performed using DNA polymerase I and RNase H. Remaining overhangs were converted into blunt ends via exonuclease/polymerase activities and enzymes were removed. After adenylation of 3’-terminal ends of DNA fragments, Illumina PE adapter oligonucleotides were ligated to prepare for hybridization. In order to select cDNA fragments of preferentially 200 bp in length, the library fragments were purified with AMPure XP system (Beckman Coulter, Beverly, USA). DNA fragments with ligated adaptor molecules on both ends were selectively enriched using Illumina PCR Primer Cocktail in a 10 cycle PCR reaction. Products were purified (AMPure XP system) and quantified using the Agilent high sensitivity DNA assay on the Agilent Bioanalyzer 2100 system. The clustering of the index-coded sample was performed on a cBot Cluster Generation System using TruSeq PE Cluster Kit v3-cBot-HS (Illumia) according to the vender’s instructions. After cluster generation, the library preparation was sequenced on an Illumina Hiseq 2000 platform and 100 bp paired-end reads were generated.

### Data filtering and de novo assembly

Image data output from the sequencing device were transformed into raw reads and stored in FASTQ format. Clean reads were obtained after removing reads that contained adaptor sequences, reads in which more than 10% of the bases were unknown, and reads in which more than half of the quality values of the bases were less than 5. *De novo* assembly was carried out using Trinity [74]. Resulting unigenes were analyzed by searching the Nr, Nt, Pfam, KOG/COG, Swiss-Prot, KEGG, and GO databases. If results obtained using different databases conflicted with each other, the sequence direction of unigenes was determined by a priority order of NR, Swiss-Prot, KEGG, and KOG. Information obtained from BLAST was used to extract CDS from unigene sequences and translate them into peptide sequences. Unigenes with no hits in BLAST were analyzed with ESTScan [75] to predict their coding regions and determine their sequence direction.

### Gene annotation and function classification

Unigenes were annotated and proteins were predicted using the nucleotide (Nt) and protein (Nr, Pfam, and Swiss-Prot) databases and assigned to functional categories in the KOG/COG, GO, and KEGG databases by BLASTx searches with E value cutoff of 10^-5^. With Nr annotation, unigenes were assigned by the Blast2GO program [53, 76] to molecular function, biological process and cellular component ontologies of the GO database (http://www.Geneontology.org). Unigenes were also searched against the KOG/COG database to predict and classify possible functions. Pathway assignments were carried out according to the Kyoto Encyclopedia of Genes and Genomes pathway database [56] also using BLASTx with E value threshold of 10^-5^.

### Data analysis

All experiments were carried out at least three times with similar results. Quantitative data were analyzed by ANOVA and Fisher’s Least Significant Difference (LSD) test.

### Availability of supporting data

The tobacco transcriptome data are available at http://www.ncbi.nlm.nih.gov/sra/?term=SRX363387.

## Electronic supplementary material

Additional file 1: Figure S1: Comparison of the *wild-type* (*WT*) tobacco plant and the transgenic control line *WT RFP* in terms of growth and development processes. (DOC 4 MB)

Additional file 2: Figure S2: Real-time RT-PCR analyses of tobacco genes involved in the anthocyanin biosynthesis pathway and expressed in flowers at developmental stages S1-S5. (DOC 418 KB)

Additional file 3: Table S1: Genes identified in the tobacco transcriptome and annotated as putative *ARF* genes by searches against seven databases. (XLS 98 KB)

Additional file 4: Table S2: Information on real-time RT-PCR analyses of putative *ARF* genes identified in the tobacco transcriptome. (DOC 88 KB)

Additional file 5: Table S3: Information on genes identified previously and primers used in this study. (DOC 44 KB)

## Abbreviations

ARF: AUXIN RESPONSE FACTOR
BLAST: Basic local alignment search tool
EST: Expressed sequence tag
GO: Gene ontology
KEGG: Kyoto encyclopedia of genes and genomes database
KOG/COG: EuKaryotic ortholog groups/clusters of orthologous groups of proteins
Nr: NCBI Non-redundant protein sequences
Nt: NCBI Nucleotide sequences
Pfam: Protein family
P35S: TTG2:RFP: The transgenic plant overexpressing the NtTTG2:RFP fusion gene under control by the cauliflower mosaic virus 35S promoter
RT-PCR: Reverse transcriptase-polymerase chain reaction
TTG: TRANSPARENT TESTA GLABRA
TTG2RNAi: The NtTTG2 silencing transgenic plant
WT RFP: The transgenic plant expressing the RFP gene coding for red-fluorescent protein.
