# Supplementary material for: TTG2-regulated development is related to expression of putative AUXIN RESPONSE FACTOR genes in tobacco
Source: BMC Genomics. 2013 Nov 20;14(1):806. doi: 10.1186/1471-2164-14-806 (PMC4046668; doi:10.1186/1471-2164-14-806)
Supplement: Supplementary file 1 — Additional file 1: Figure S1: Comparison of the wild-type (WT) tobacco plant and the transgenic control line WT RFP in terms of growth and development processes. (DOC 4 MB) [file 12864_2013_5526_MOESM1_ESM.doc]

**
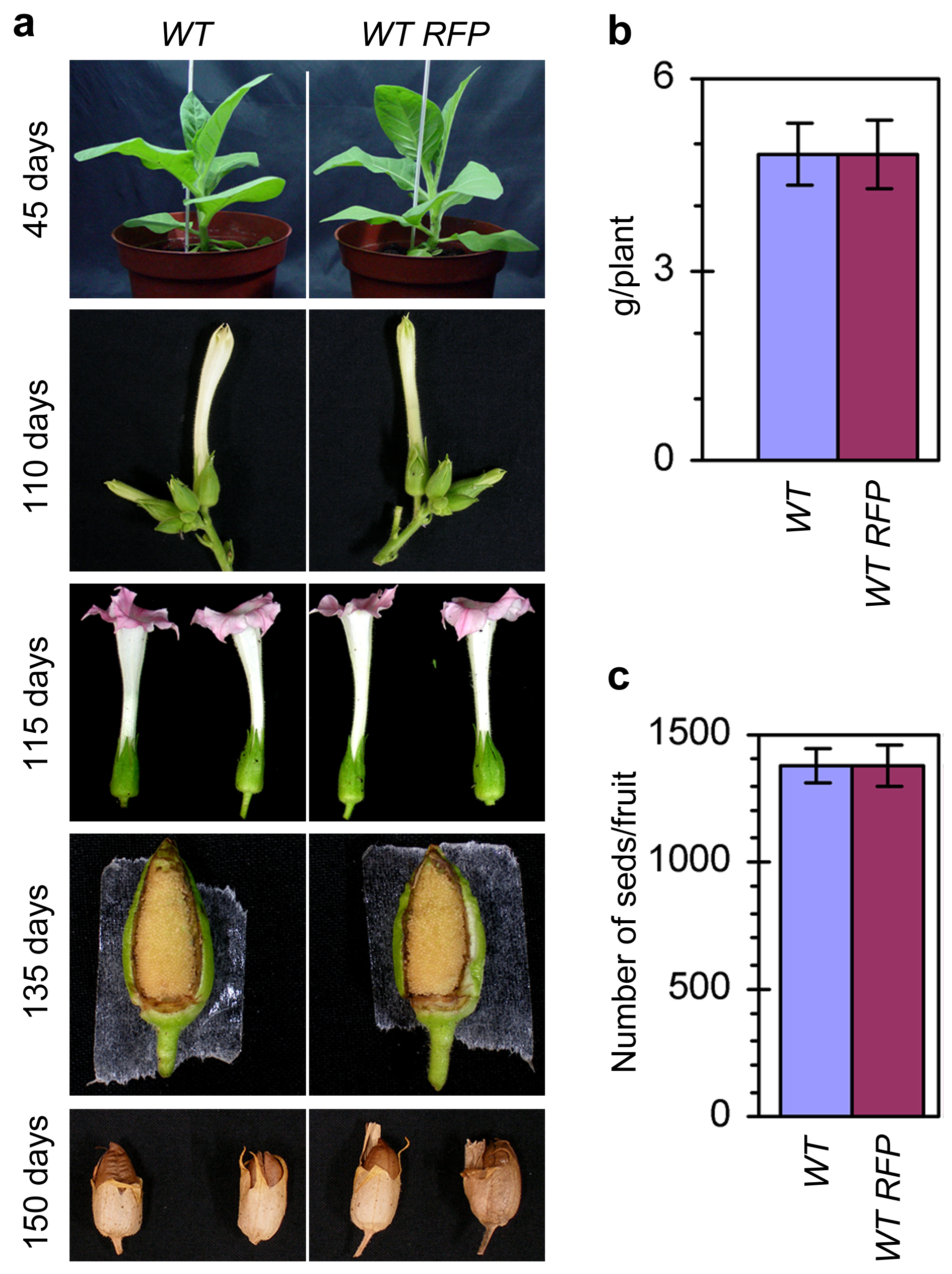
**

**Additional file 1: Figure S1.** Comparison of the *wild-type* (*WT*) tobacco plant and the transgenic control line *WT RFP* in terms of growth and development processes.

The tobacco *Nicotiana tabacum* variety NC89 was used as the transformation host. The transgenic control line *WT RFP* was generated by transformation of NC89 with a plant binary vector that carried out an insert of the *RFP* gene encoding red-fluorescent protein. **(a)** Monitoring of the vegetative growth and flower and fruit development. **(b)** Fresh weight of 45-day-old plants. **(c)** Seed count. Data shown in (b,c) are mean values ± standard deviation (SD) bars of results from seven experimental repeats (50 plants/repeats).
