# Supplementary material for: TTG2-regulated development is related to expression of putative AUXIN RESPONSE FACTOR genes in tobacco
Source: BMC Genomics. 2013 Nov 20;14(1):806. doi: 10.1186/1471-2164-14-806 (PMC4046668; doi:10.1186/1471-2164-14-806)
Supplement: Supplementary file 2 — Additional file 2: Figure S2: Real-time RT-PCR analyses of tobacco genes involved in the anthocyanin biosynthesis pathway and expressed in flowers at developmental stages S1-S5. (DOC 418 KB) [file 12864_2013_5526_MOESM2_ESM.doc]

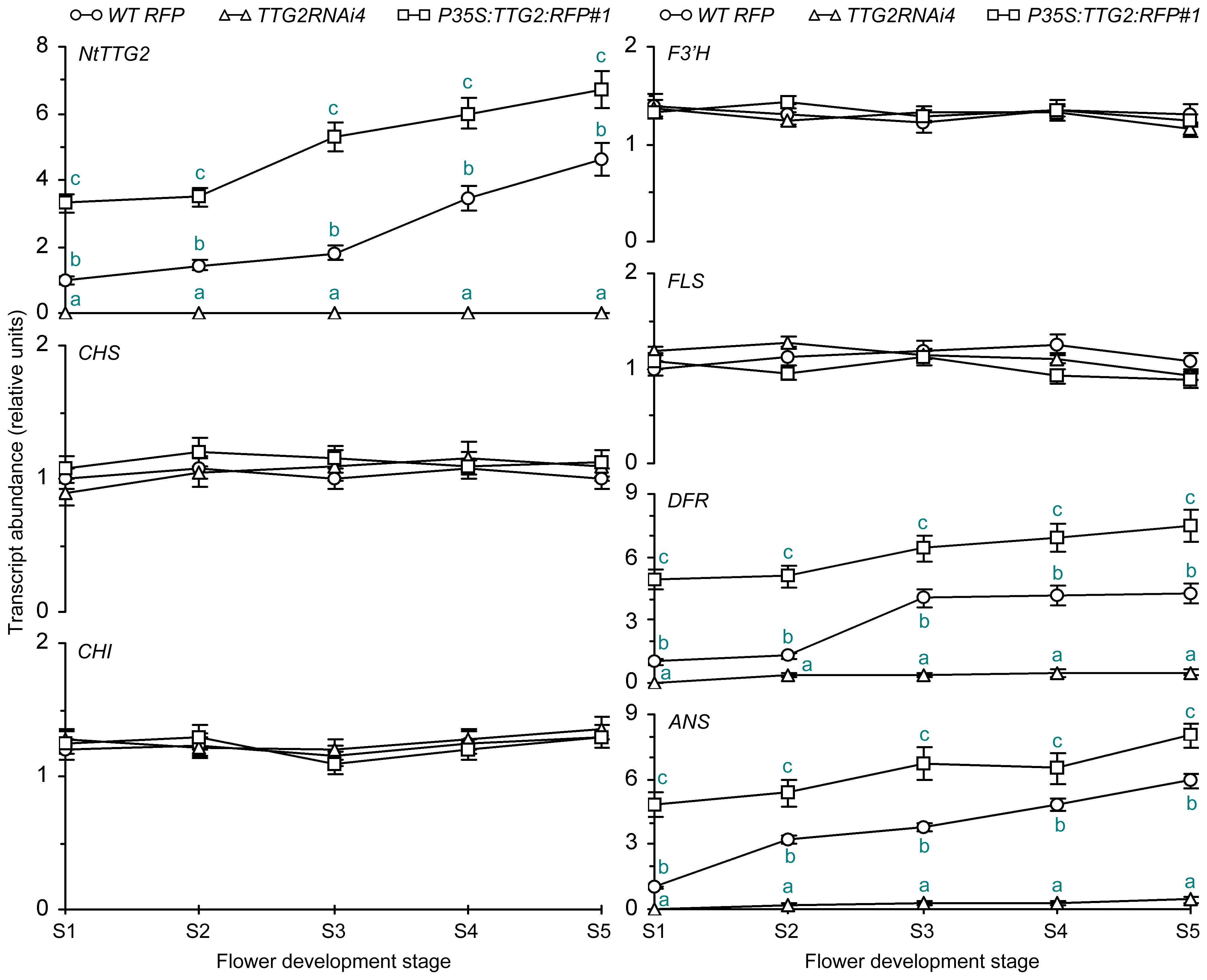


**Additional file 2: Figure S2.** Real-time RT-PCR analyses of tobacco genes involved in the anthocyanin biosynthesis pathway and expressed in flowers at developmental stages S1-S5.

Data shown are mean values ± SD bars of results from three experimental repeats (15 flowers/repeat). Different letters on the graphs indicate significant differences analyzed by one-way ANOVA and LSD test of data from the same stage of flower development.
