# Supplementary material for: TTG2-regulated development is related to expression of putative AUXIN RESPONSE FACTOR genes in tobacco
Source: BMC Genomics. 2013 Nov 20;14(1):806. doi: 10.1186/1471-2164-14-806 (PMC4046668; doi:10.1186/1471-2164-14-806)
Supplement: Supplementary file 4 — Additional file 4: Table S2: Information on real-time RT-PCR analyses of putative ARF genes identified in the tobacco transcriptome. (DOC 88 KB) [file 12864_2013_5526_MOESM4_ESM.doc]

**Additional file 4: Table S2.** Information on real-time RT-PCR analyses of *ARF* candidates identified in the tobacco transcriptome

| Unigene (*ARF* candidate) ID | Primers | Product length (bp) |
| --- | --- | --- |
| comp1238_c0 | 5’-CATTGCAGCATTCACAGAAGACG-3’,  5’-GCAGGGTAAAGCGGGGTGGTATT-3’ | 203 |
| comp19484_c2 | 5’-GGAGAGCCATAACCAAAGCGAC-3’  5’-AGGCTAGCTGATGTAATCTGAGACTG-3’ | 207 |
| comp26539_c0 | 5’-CTGCTTTGCGTTTTCCATCGTG-3’  5’-CTCCGCAAGAAGTCCAGCAAATG-3’ | 227 |
| comp30272_c0 | 5’-TTGTTTACTTTCCTCAGGGTCAT-3’  5’-GTGTCACTTGCAGTCAGCGTC-3’ | 188 |
| comp31238_c0 | 5’-GATGTTCTCCTCCTCGGTGAC-3’  5’-ATTTCGGGAGCCCTTTTGG-3’ | 201 |
| comp31531_c0 | 5’-CAAGGGCGGTAGTAAATGGTGA-3’  5’-GGTTTGTAAGTGCGAAGAAGC-3’ | 221 |
| comp38086_c0 | 5’-ATCAGCCTAAGTGTTGCCGTTGT-3’  5’-TTTACTTGCTCGGACAGTTCACC-3’ | 220 |
| comp38146_c2 | 5’-GATGAGTCAACTGCTGGGGAGAG-3’  5’-AAAGTCATCACCAAGCCACGG-3’ | 198 |
| comp39443_c0 | 5’-GAGGGTTAAGATGGCTGTGGAGACG-3’  5’-GTGAAGCTGCGGGATTGGAAGAATA-3’ | 214 |
| comp40625_c0 | 5’-AATACCGACTCTTTGAACATACTGC-3’  5’-GTTCTATTAGTTGGGTTTGCTTGG-3’ | 225 |
| comp41729_c1 | 5’-CTGGAGTTCTGTAAGATGGTCAGGAGG-3’  5’-GAAAAAGCACGTCTATTCGTTAAGCC-3’ | 228 |
| comp41729_c2 | 5’-AGTAAGCAGGTGTCTTCGAGGTTGG-3’  5’-CATGCGGTTCATTCGTTCTGTAAAA-3’ | 216 |
| comp42904_c0 | 5’-TCAGGGCTTCTCCTCCCGACA-3’  5’-TCCAGTGACCTCCCAACAGACC-3’ | 224 |
| comp15074_c0 | 5’-ACAGGCTGCTGAAACTGCATTAAC-3’  5’-GTGAGCTAGGACCTCATTCGGTG-3’ | 190 |
| comp153585_c0 | 5’-ATCGTACGAGGCGTTACACAAG-3’  5’-TGCTGCTTGCATCCGTTAAA-3’ | 179 |
| comp19484_c1 | 5’-GGGATGTGGAGACAAGCAGAGTAG-3’  5’-TGACCATAGGTTTGAGATTGAGGAA-3’ | 223 |
| comp2000_c0 | 5’-AGCTGCTTAGAGAGATCTGCTGTTC-3’  5’-CATTCAAGTCCGTTTCCTGTCG-3’ | 232 |
| comp218520_c0 | 5’-TAATGAAGAGGGGAAAAGTGAAG-3’  5’-TCATCGTTGAGTCTTCAGTTTCG-3’ | 210 |
| comp23738_c0 | 5’-TGTTTGTGTTTCCTTACGTTTCG-3’  5’-GCATGCTCAGTGTGGATTATCG-3’ | 223 |
| comp24631_c0 | 5’-GACCTCCCAACACACCCCGATT-3’  5’-CAGGGCTTCTCCTTCCAATCAC-3’ | 214 |
| comp25452_c0 | 5’-GAGGGGACACTACGATGACAACT-3’  5’-ACATGCACGGACTGCCAGAT-3’ | 207 |
| comp25452_c1 | 5’-TTGTTGAACTGGAGACCTGGTGTAG-3’  5’-ATTAAAGAGGGGGATGCCTTGGAT-3’ | 228 |
| comp25955_c0 | 5’-GTGCATTGGGATGAGCCAGATT-3’  5’-GCTTAAAGGGTTGTTGAGAAGTGA-3’ | 199 |
| comp25955_c1 | 5’-TTGAGTTCAGTGGTGGCATTTGG-3’  5’-CATGGAAGCTCGGCTTTGTATTT-3’ | 225 |
| comp27156_c0 | 5’-CTCCAACCAGTTGTCAACAAATGCC-3’  5’-CAAATAACGGGGGTGGTTTTTCAGT-3’ | 184 |
| comp27156_c1 | 5’-CCTGCTGAAGAAGTAGGAGGTGTTT-3’  5’-GTTGGAGTTTAATGGAGGAATTTGC-3’ | 193 |
| comp28806_c0 | 5’-GTCAAGTGTTCGACCTACATCCTC-3’  5’-GGCAACAGTTTATCTAATGGGAAT-3’ | 204 |
| comp34649_c0 | 5’-AGATGCTAACAAATGCTTCCCTC-3’  5’-TGTAGTTTGCCAGTTTCATCCCT-3’ | 225 |
| comp35069_c0 | 5’-GTATTCACGGAAGACGCCCTAAC-3’  5’- GAAGACATGAACAAGAACGGAAGC-3’ | 185 |
| comp38146_c0 | 5’-TCTTGGAGATGTTCACTGAGGGC-3’  5’-TTCACCATCCACGAACAACTGTC-3’ | 209 |
| comp38146_c1 | 5’-GGCACGGATTGGTTGATTGGATT-3’  5’-ACAGAATGTCGGAAGTGGAGCAG-3’ | 228 |
| comp38411_c0 | 5’-ATTCCTGGATTGGTTTCTGCTT-3’  5’-CCTGATACTGATGAGGTTTACGC-3’ | 235 |
| comp40082_c0 | 5’-TGTCACAGCAACGCCTAC-3’  5’-CCATTCTCGCCATAACCC-3’ | 225 |
| comp41091_c0 | 5’-CAAGCCGTCTAACACCAACT-3’  5’-ACCAGCAGACTCCAACTCAAGAAT-3’ | 214 |
| comp42071_c0 | 5’-TTTGCCCGCCTAATGCCTAC-3’  5’-CCTCCTGTTCAGACCATCCTTGC-3’ | 208 |
| comp42359_c0 | 5’-GTGTTCGTCGTTACATGGGC-3’  5’-CAGAGGAAATGGCGATGGATA-3’ | 196 |
| comp42359_c1 | 5’-AACAGACATGATGCCAGAG-3’  5’-GAGTTCGTCAACAGCGTAT-3’ | 185 |
| comp42388_c0 | 5’-CAAGGACAAGCCCTACCGAG-3’  5’-TCCCACCGAACCTTCAGAC-3’ | 216 |
| comp42867_c0 | 5’-CTGTGCGAGCTAGGAGTAGAGGG-3’  5’-TGGGATTAGGAGGGAAGAAGATG-3’ | 195 |
| comp43225_c0 | 5’-GAGCCTACCACAGCAACAGC-3’  5’-GATTGACCCAAGATGCGAAGA-3’ | 210 |
| X97131 (*EF1α* used as a reference gene) | 5’-TGACAAACTAAAGGCTGAGCGTGAC-3’  5’-CAAAGCCAGTGGTGGAGGCAAC-3’ | 185 |
