# Supplementary material for: TTG2-regulated development is related to expression of putative AUXIN RESPONSE FACTOR genes in tobacco
Source: BMC Genomics. 2013 Nov 20;14(1):806. doi: 10.1186/1471-2164-14-806 (PMC4046668; doi:10.1186/1471-2164-14-806)
Supplement: Supplementary file 5 — Additional file 5: Table S3: Information on genes identified previously and primers used in this study. (DOC 44 KB) [file 12864_2013_5526_MOESM5_ESM.doc]

**Additional file 5: Table S3.** Information on genes identified previously and primers used in this study

| Gene | Accession ID | Primers | Test | Product length (bp) |
| --- | --- | --- | --- | --- |
| *NtTTG2* | FJ795022 | 5’-AGCGGAGTTGGAAAGGCATCAGG-3’  5’-ATCAATCCCATTAGGCCCAGCAA-3’ | Real-time RT-PCR | 189 |
| *EXP1* | AF049350 | 5’-GCAATCCAATGCATATCTCAATGGC-3’  5’-AGTAAGCACGCCGCTGCCTATGACG-3’ | Real-time RT-PCR | 210 |
| *EXP2* | AF049351 | 5’-TGTCCACCAAATTATGCACTTCC-3’  5’-TAGTTCCTTCCATTTATTGTGAATC-3’ | Real-time RT-PCR | 191 |
| *DFR* | AB289448 | 5’-GCCGCTGGTTGTTGGTCCATTCCTC-3’  5’-CTTCCCTCTGCCTTTGGCTGCTCAT-3’ | Real-time RT-PCR | 180 |
| *ANS* | AB289447 | 5’-TTGCGTTTTCCCCGAGGACAAGTGC-3’  5’-ATTTGAAGCAGTAGGTCCTCCTTGC-3’ | Real-time RT-PCR | 201 |
| *CHS* | EU503226 | 5’-AGCCGCGATCATTATAGGTTCTG-3’  5’-TGGAATGCTTCTATTAGGCTCTTC-3’ | Real-time RT-PCR | 207 |
| *CHI* | AB213651 | 5’-AAATAGAGGTTTGGAAATTGAAGGG-3’  5’-ACCCGTCAAAGGCAAGATCATAG-3’ | Real-time RT-PCR | 208 |
| *F3’H* | AF036093 | 5’-CTTCAAGCGACTCGTGATGGTGG-3’  5’-GTACACAATTGCGTTTGGTGCAGGG-3’ | Real-time RT-PCR | 204 |
| *FLS* | AB289451 | 5’-CAGGCAATAGCGTCAATAACAAA-3’  5’-TATCACTTGAAATATCCCCCATTCT-3’ | Real-time RT-PCR | 192 |
| *EF1α* | X97131 | 5’-TGACAAACTAAAGGCTGAGCGTGAC-3’  5’-CAAAGCCAGTGGTGGAGGCAAC-3’ | Real-time RT-PCR | 185 |
